# Assessment of School-Based Quasi-Experimental Nutrition and Food Safety Health Education for Primary School Students in Two Poverty-Stricken Counties of West China

**DOI:** 10.1371/journal.pone.0145090

**Published:** 2015-12-14

**Authors:** Minxue Shen, Ming Hu, Zhenqiu Sun

**Affiliations:** Department of Epidemiology and Health Statistics, School of Public Health, Central South University, Changsha, Hunan, China; Chiba University Center for Forensic Mental Health, JAPAN

## Abstract

**Background:**

Few studies on nutrition and food safety education intervention for students in remote areas of China were reported. The study aimed to assess the questionnaire used to measure the knowledge, attitude and behavior with respect to nutrition and food safety, and to evaluate the effectiveness of a quasi-experimental nutrition and food safety education intervention among primary school students in poverty-stricken counties of west China.

**Methods:**

Twelve primary schools in west China were randomly selected from Zhen’an of Shaanxi province and Huize of Yunnan province. Six geographically dispersed schools were assigned to the intervention group in a nonrandom way. Knowledge, attitude and behavior questionnaire was developed, assessed, and used for outcome measurement. Students were investigated at baseline and the end of the study respectively without follow-up. Students in intervention group received targeted nutrition and food safety lectures 0.5 hour per week for two semesters. Item response theory was applied for assessment of questionnaire, and a two-level difference-in-differences model was applied to assess the effectiveness of the intervention.

**Results:**

The Cronbach’s alpha of the original questionnaire was 0.84. According to item response model, 22 knowledge items, 6 attitude items and 8 behavior items showed adequate discrimination parameter and were retained. 378 and 478 valid questionnaires were collected at baseline and the end point. Differences of demographic characteristics were statistically insignificant between the two groups. Two-level difference-in-differences models showed that health education improved 2.92 (95% CI: 2.06–3.78) and 2.92 (95% CI: 1.37–4.47) in knowledge and behavior scores respectively, but had no effect on attitude.

**Conclusion:**

The questionnaire met the psychometric standards and showed good internal consistence and discrimination power. The nutrition and food safety education was effective in improving the knowledge and behavior of primary school students in the two poverty-stricken counties of China.

## Introduction

While child and adolescent obesity has become a significant global health problem [1, 2] and the distribution of nutrition-related diseases is shifting from a predominance of undernutrition to a dual burden of malnutrition and overnutrition in low- and middle-income countries [3], malnutrition in childhood, which is estimated to cause 3.1 million child deaths annually through a potentiating effect on common infectious diseases such as pneumonia and diarrhea [4], remains unoptimistic. Globally, underweight prevalence decreased from 25% in 1990 to 15% in 2012, which remains insufficient to meet Millennium Development Goal of halving the 1990 prevalence by 2015; and 67% of all underweight children lived in Asia [5]. China is undergoing sharp economic development during the past 30 years and childhood nutrition has been improved greatly, accompanied by an increased prevalence of obesity [6, 7]. However, the prevalence of malnutrition problems such as anemia, vitamin A deficiency and growth and development retardation among children in rural areas are much greater than those in urban, especially in western China [8, 9]. The prevalence of child underweight and growth retardation in rural China was 6.1% and 16.3% respectively in 2005 [10].

The KAP (knowledge, attitude and practice) model is the theoretical foundation of most health education programs [11]. According to this model, adequate eating behavior (food selection and recognition) occurs due to healthy attitude, which in turn develops due to proper knowledge on nutrition and food safety. Most of the previous studies that were conducted in developed countries emphasized fruit and vegetable intake, owing to an increasing rate of obesity among children. Lakshman et al. conducted a game-oriented intervention on primary school students in UK and improved their nutrition knowledge compared with the control group [12]. Wall et al. observed improved vegetable-related attitude, knowledge and self-efficacy of fourth grade students after a 4-lesson classroom-based nutrition education program [13]. Some studies indicated that health education alone was insufficient to change student’s practice patterns in short-term observations [14–16]. In China, studies of nutrition education for young children were rarely reported. Zhou et al. observed improved nutrition and food safety knowledge among primary and junior school student in Chongqing, China after 9-month health education, through a school-based cluster trial [17]. However, the education was not designed for students in remote areas. A remote area is one that is either a long distance from highly populated settlements or which lacks transportation links that are typical in more populated areas. In China, these areas are characterized by limited accessibility of clean water and fresh foods, maldistribution of teachers and health practitioners, high rate of left-behind children, and high prevalence of childhood malnutrition. Children in remote areas have limited intake of both fresh vegetables and meats, and they are facing a higher risk of food poisoning caused by eating wild foods. In addition, the development and assessment of the research tool in some of the previous studies were unclear, and the statistical approaches were not capable of treating repeatedly measured data.

Owing to the absence of study that focused on specific nutrition education for children in remote areas, as well as the inappropriate use of statistical methods in program evaluation, we conducted this quasi-experimental nutrition and food safety education program among primary students in Shaanxi and Yunnan provinces for two goals: (1) to evaluate the reliability of the questionnaire based on classical and modern test theory; (2) to assess the effectiveness of the school-based education program in remote areas of China.

## Materials and Methods

### Study design

**Fig 1:**
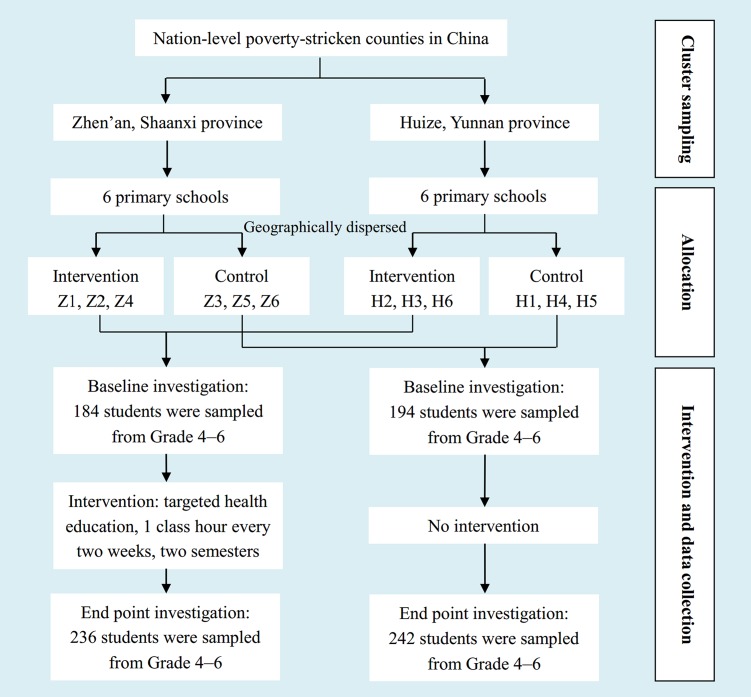
Research process flow chart.

The study aimed to assess the reliability of the knowledge, attitude and behavior of nutrition and food safety questionnaire for primary school students (Grade 4 to 6) in poverty-stricken counties of China, and evaluate the effectiveness of health education through a quasi experiment, in order to promote policy establishment for child and adolescent health in the future. Two nation-level poverty-stricken counties (defined by area-specific annual income per capita, and announced by Chinese government), Zhen’an and Huize were randomly selected from Shaanxi (northwest China) and Yunnan (southwestern China) respectively. Twelve schools were randomly selected from the two counties (six in each county) using a random number table. We assigned six geographically dispersed schools to the intervention group in a nonrandom way (Z1, Z2 and Z4 from Zhen’an, and H2, H3 and H6 from Huize), as shown in Fig 1. The other six schools were assigned to the control group.

Sample size was estimated as the following. Assuming that the post-intervention difference (score) between intervention and control group = 2.0 with a variance = 10.0, an intra-cluster correlation coefficient = 0.15, alpha = 0.05, beta = 0.20, and average size of each cluster = 40, a total of 198 students in each group were required. Our results indicated that the statistical power had exceeded 80%.

### Data collection

Data collection included two stages: baseline and final investigation. Ten to fifteen students were randomly selected from a class in Grade 4, 5 and 6 respectively, through a systematic sampling procedure according to the students’ ID number, resulting in thirty to forty five students in each school. Baseline data collection was conducted in Sep 2010, and end point survey was conducted in May 2012. Follow-ups and repeated measures were not applicable in our research because students from grade 6 in 2010 had graduated from primary school, and follow-up was impossible. As a result, our sample at baseline and final survey were two different cohorts.

During data collection, students from the same school were assembled in a classroom to complete the self-administered questionnaires. Investigators were responsible for explaining the details to students to ensure that they understand all items, and examining the completeness of recycled questionnaires In the final survey, we were unable to identify students who had not received educational courses due to absenteeism, sickness or other uncontrolled conditions.

### Research tools and outcome measurement

We used *The Knowledge*, *Attitude and Behavior Questionnaire on Nutrition and Food Safety for Students in Grade 4 to 6* as the tool for outcome measurement.

The questionnaire was originally designed by nutritionists and experts of school health in Central South University. Then the questionnaire was revised and improved using a Delphi method. The Delphi panel consisted of 25 experts including nutritionists (n = 6), experts of school health (n = 2), biostatisticians (n = 8), epidemiologists (n = 2), and officials of educational departments (n = 7). Through three rounds of consultations and feedbacks, an initial pool of 72 items was derived. Content validity of the questionnaire was evaluated by the Delphi panel. Then, a pilot test was conducted in 120 students in two primary schools in Ningxiang, Hunan province. The questionnaire items were then selected by statistical methods as follows: (1) Student’s *t* test. Subjects were ranked by the score on the scale to derive a high- and low-score group, comprising 27% of those with the highest and lowest scores, respectively. The score of each item was then compared using t-tests. Items with no significant difference (*P*<0.05) between the groups were eliminated. (2) Pearson’s correlation coefficient. Any item with a coefficient <0.30 with the total scale score was eliminated. After the pilot test, a 54-item questionnaire with good reliability was derived. Content validity was evaluated by the Delphi panel.

This questionnaire included demographic information (grade, age, gender, height, weight, parents’ education levels, number of siblings, left-behand child), 31 items of knowledge (true or false and single-answer questions), 7 items of attitude (single-answer questions) and 16 items of behavior (single-answer questions). Examples of items are shown in Table 1.

**Table 1:** Examples of items.

Dimension	Issue	Type of items	Examples
Knowledge	Nutrition	True-or-false	K03. We should eat sufficient cereal food.
Knowledge	Food safety	True-or-false	K04. Uncooked or undercooked kidney bean is not edible.
Knowledge	Nutrition	Single-answer	K25. Which of the following foods contains most vitamin C: (1) Soybean. (2) Orange. (3) Milk. (4) Pork. (5) I do not know.
Attitude	Nutrition	Single-answer	A03. If you realize that your eating pattern is not healthy, you would like to change it immediately: (1) Strongly agree. (2) Agree. (3) Not sure. (4) Disagree. (5) Strongly disagree.
Attitude	Food safety	Single-answer	A05. You would like to participate in the lecture on food safety that is held on your campus: (1) Strongly agree. (2) Agree. (3) Not sure. (4) Disagree. (5) Strongly disagree.
Practice	Nutrition	Single-answer	P04. How often did you eat breakfast: (1) Never. (2) Once or twice per week. (3) Three or four times per weeks. (4) Five or six times per week. (5) Every day.
Practice	Food safety	Single-answer	P12. Did you pay attention to the expiration date printed on food package when purchasing: (1) Never. (2) Seldom. (3) Occasionally. (4) Often. (5) Always.

### Intervention

The basic intervention strategy was targeted health education on nutrition and food safety, developed by experts of adolescent health, nutrition and public health from Central South University, officials from Chinese Ministry of Education and UNESCO, and local officials. Course syllabus and textbook for students in Grade 4 to 6 were designed and revised during Nov 2010 and Jun 2011. Biology teachers in primary schools were designated as the course givers and were trained systematically by our professionals for one week. The intervention was implemented from Sep 2011 to May 2012 for all students in intervention group: 0.5 hour per week for two semesters (sharing the class with the routine health education).

The *Nutrition and Food Safety Textbook for Students in Grade 4 to 6*, was designed according to the baseline information and regional characteristics in west China. Except general nutrition and food safety knowledge (category of food, food pyramid, nutrient, vitamins and minerals, habit of drinking water, identification of food packaging, obesity, etc.), several issues were specifically emphasized in our intervention, according to the problems revealed in baseline investigation: (1) Diversity of foods. Potato and corn were the main food sources among these children, while fresh vegetables, meats and beans were rarely consumed. (2) Water consumption. Drought has long been a severe problem in Yunnan province. Although the government built cisterns for schools, rain water was collected for drinking without purification. (3) Breakfast. The schools locate in remote mountainous areas and food was expensive owing to transportation cost. Most students never ate breakfast due to economic concern. (4) Food poisoning caused by eating wild foods (e.g. poisonous mushrooms). (5) Potential food safety problems of snacks. Some snacks sold by stories near the schools were made by family workshops without production permits.

Multimedia equipment (laptops and projectors), electronic teaching materials, wall maps and food cards were provided for each of the six schools. Students were given edutainment-oriented lectures, learning with vivid and interesting figures, examples and stories. Broadcast and bulletin in schools were also used for educational purpose. In order to supervise the quality of course, we examined the curriculum schedule, teaching plan and student homework, and attended open classes during the period. Students in control group did not receive any intervention from the researchers.

### Statistical approach

Item response theory (IRT) was used to evaluate the precision of the measurements. IRT is a family of associated mathematical models that relate latent traits (ability) to the probability of responses to items in an assessment, and it has been widely used in psychometrics and health assessment [18, 19]. It specifies a nonlinear relationship between binary, ordinal, or categorical responses and the latent trait (KAP in this case). Compared with classical test theory approaches, the advantages of IRT include near-equal interval measurement, representation of respondents and items on the same scale; and independence of person estimates from the particular set of items used for estimation [20]. Eq (1) specifies a two-parameter logistic IRT model, where *P* is the probability of correct response (i.e. *Y* = 1), *θ*_*i*_ is the ability of the participant *i*, *α*_*k*_ is the discrimination parameter of the item *k*, and *b*_*k*_ is the difficulty parameter of the item *k*. The difficulty parameter is the point on the ability scale that corresponds to a probability of a certain response of 50%; the discrimination parameter estimates how well an item can differentiate among respondents with different levels of ability. Eq (2) specifies a polytomous IRT model, which is used for items with multiple categories (e.g. Likert-type). In this model, the probability of scoring in a specific category is modeled by the probability of responding in this category minus the probability of responding in the next category. *κ*_*k*,*c*_ is the upper grade threshold parameter for category *c*.

$$P(Y_{i}=1|\theta_{i},\alpha_{k},b_{k})={\left[1+\exp\left(b_{k}-\alpha_{k}∙\theta_{i}\right)\right]}^{-1}$$ (1)

$$P(Y_{ik}=c|\theta_{i},\kappa_{k})=\Phi (\kappa_{k,c}-\alpha_{k}{∙\theta }_{i})-\Phi (\kappa_{k,c-1}-\alpha_{k}∙\theta_{i})$$ (2)

In this study, IRT was applied for items selection. Two-parameter logistic model was used to fit the binary items, and generalized partial credit model was used to fit graded items (Likert-type questions). IRT parameters were estimated using a marginal maximum likelihood method. The criteria for item deletion was: 1) discrimination parameter < 0.5 or > 2.0; or 2) difficulty parameter < -3.0 or > 3.0 [18, 21, 22].

The government of Shaanxi Province initiated the EGG & MILK PROJECT for students that receive compulsory education since Sept 2009, providing each student one egg and 200 ml milk or soymilk every day. Therefore, we also deleted items on egg, milk and bean products intake in behavior dimension, in order to avoid unexpected influence. Guessing parameter was not included in IRT model because the “*I do not know*” choice was included in all knowledge questions.

Chi-square tests were used to compare demographic variables between groups and one-way ANOVA was used to compare average scores between groups.

Owing to the structured and repeated cross-sectional data, multilevel statistical models and difference-in-differences (DID) models were combined to estimate the effect of intervention and intra-cluster correlation. In Eq (3), *G* is the group variable (intervention vs. control), *T* is the time variable (pre- vs. post- intervention), *G*·*T* is the interaction between group and time (i.e. the DID estimator), *X*_*i*_ refers to the cofounding factors, *ν*_*gt*_ and *u*_*igt*_ are the random errors at school and individual level, respectively.

$$Y_{i}=\alpha ∙T+\lambda ∙G+\gamma ∙G∙T+\beta_{i}∙X_{i}+\upsilon_{gt}+u_{igt}$$ (3)

Goodness of fit of null models were compared so that the number of levels could be determined. Dimension scores were calculated separately, because scoring methods were different.

IRT parameters were estimated using PASCALE 4.1 (Scientific Software International Inc., Lincolnwood). Multilevel DID models were estimated using MLwiN 2.1 (Rasbash J, Charlton C, Browne WJ, Healy M and Cameron B, Centre for Multilevel Modelling, University of Bristol). Other analyses were performed using SAS 9.4 (SAS Institute Inc., Cary, North Carolina). The significance level of all statistical tests was 0.05.

### Ethics statement

The research protocol has been reviewed and approved by the Ethics Committee of Central South University. We obtained written informed consents from all parents or main caregivers of the enrolled children through parent-teacher conferences. The purpose of the study and details of the intervention were explained by our research group, and the implication of the program was introduced by officials of the local education department. We also obtained written informed consents from all biology teachers who gave the nutrition and food safety courses to the students in the intervention group.

## Results

### Assessment of the questionnaire

**Fig 2:**
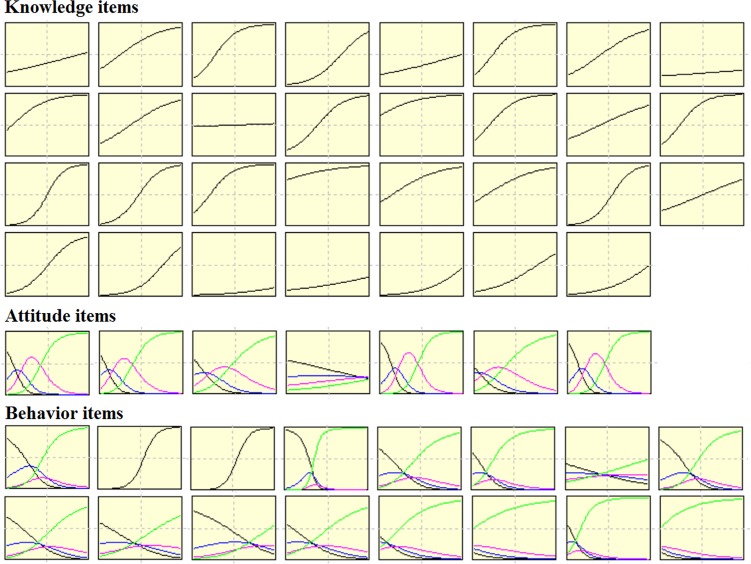
Item characteristic curves. Each Item characteristic curve describes the item-specific relationship between the ability level (X-axis) and probability of the ‘correct’ response (Y-axis). Ability in the item response theory model practically (though not exclusively) ranged from −3 to +3. The difficulty parameter is the point on the ability scale that corresponds to a probability of a correct response of 50%. The discrimination parameter is the slope of each curve. For Likert-type attitude and practice items, polytomous item response model were applied (multiple curves within a single figure, each curve stands for the relationship between ability and probability of a certain response).

**Fig 3:**
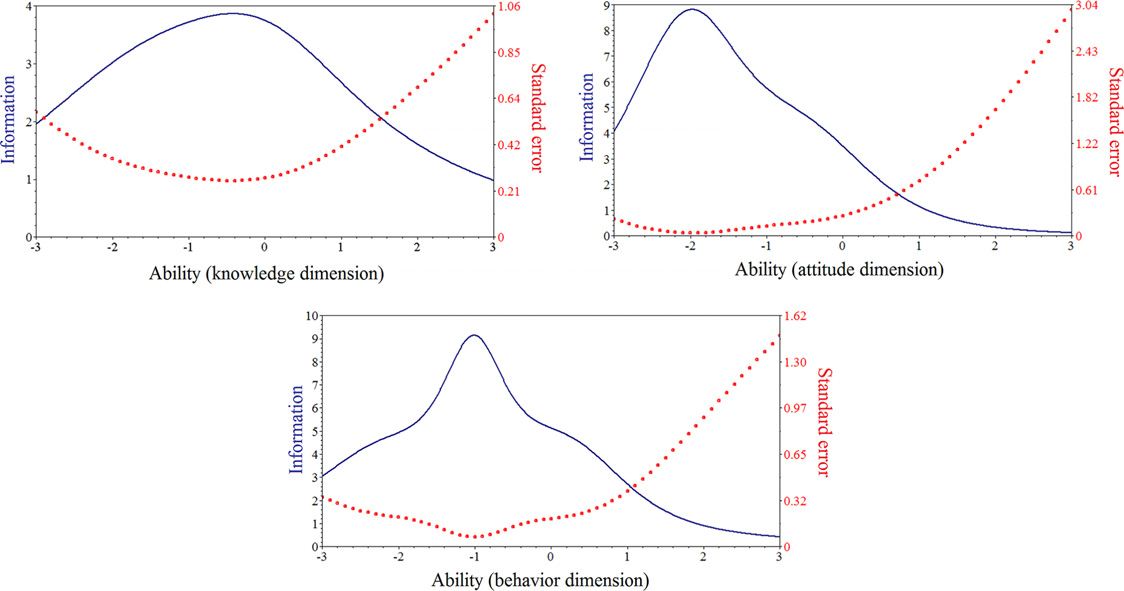
Test information curves. Ability signifies knowledge, attitude and behavior with respect to nutrition and food safety, estimated using the maximum-likelihood method. Ability in the item response theory model practically (though not exclusively) ranged from −3 to +3. The test information of knowledge dimension reached a peak when the ability was between 0 and 1; this indicates that the measurement exhibited highest discriminative power among students with moderate ability with respect to nutrition and food safety knowledge. By contrast, this questionnaire exhibited highest discriminative power among students with limited ability with respect to attitude and behavior.

The Cronbach’s alpha was 0.84 for the original questionnaire, and the test-retest reliability was 0.83. Items were selected according to IRT parameters as defined in method section. Item characteristic curves were shown in Fig 2. Nine items of knowledge, one item of attitude, and eight items of behavior showed insufficient discrimination power and were removed from the original questionnaire. The test information curves were shown in Fig 3. Test information of knowledge items peaked among students with moderate ability, while attitude and behavior items had stronger discriminative power among students with limited ability. Finally, 22 items of knowledge, 6 items of attitude and 8 items of behavior were selected from the original questionnaire. The Cronbach’s alpha was 0.80 for the questionnaire with selected items.

### Description of baseline and final survey

378 (94.5%) and 478 (95.6%) valid questionnaires were collected from baseline and final survey respectively. Demographic information of the students are shown in Table 2, and no statistical significances were found between the groups. Differences of knowledge, attitude and behavior scores between the intervention and control group were insignificant at baseline, and became significant after intervention. Scores in end point increased in both groups compared with baseline (Table 3).

**Table 2:** Demographic information of randomly sampled students.

	Baseline		End point	
	Intervention	Control	Intervention	Control
N	184	194	236	242
Age	10.80±1.14	10.91±1.25	11.70±1.22	11.72±1.15
BMI *	15.83±2.95	16.49±4.32	16.47±2.79	17.58±9.27
Grade				
4	64 (34.8)	68 (35.1)	76 (32.2)	79 (32.6)
5	61 (33.2)	66 (34.0)	81 (34.3)	82 (33.9)
6	59 (32.1)	60 (30.9)	79 (33.5)	81 (33.5)
Gender				
Male	80 (43.5)	87 (44.8)	107 (45.3)	105 (43.4)
Female	104 (56.5)	107 (55.2)	129 (54.7)	137 (56.6)
Number of siblings				
0	37 (20.1)	40 (20.6)	46 (19.5)	36 (14.9)
≥ 1	147 (79.9)	154 (79.4)	190 (80.5)	206 (85.1)
Left-behind children ^†^				
Yes	50 (27.2)	45 (23.2)	57 (24.2)	65 (26.9)
No	134 (72.8)	149 (76.8)	179 (75.8)	177 (73.1)
Father’s educational level				
Primary school or illiterate	71 (38.6)	82 (42.2)	91 (38.6)	96 (39.7)
Junior school	63 (34.2)	56 (28.9)	94 (39.8)	80 (33.1)
High school or collage	16 (8.7)	12 (6.2)	17 (7.2)	16 (6.6)
Unknown / Not applicable	34 (18.5)	44 (22.7)	34 (14.4)	50 (20.6)
Mother’s educational level				
Primary school or illiterate	64 (34.8)	58 (29.9)	73 (30.9)	76 (31.4)
Junior school	78 (42.4)	78 (40.2)	109 (46.1)	99 (40.9)
High school or collage	21 (11.4)	26 (13.4)	27 (11.5)	37 (15.3)
Unknown / Not applicable	21 (11.4)	32 (16.5)	27 (11.5)	30 (12.4))

BMI (body mass index) = height (m) / [weight (kg)]^2^

* BMI was self-reported and might not be accurate.

† Left-behind children refers to children whose parents leave for working for consecutive 6 months or longer. These children are usually under the care of relatives, mostly grandparents with very limited education.

**Table 3:** Dimension scores before and after educational intervention.

	Intervention Group		Control Group		*P*
	N	Mean (95% CI)	N	Mean (95% CI)	
Baseline					
Knowledge *	184	11.4 (10.9, 11.8)	194	11.4 (10.9, 11.8)	0.953
Attitude ^†^	184	26.0 (25.6, 26.5)	194	26.4 (25.9, 26.8)	0.384
Behavior ^‡^	184	26.3 (25.3, 27.3)	194	26.9 (25.9, 28.0)	0.379
End point					
Knowledge *	236	16.0 (15.7, 16.4) ^§^	242	13.7 (13.3, 14.1) ^§^	<0.001
Attitude ^†^	236	27.8 (27.5, 28.0) ^§^	242	26.8 (26.5, 27.2)	<0.001
Behavior ^‡^	236	32.0 (31.3, 32.8) ^§^	242	29.3 (28.5, 30.1) ^§^	<0.001

CI: confidence interval

* The knowledge dimension included 22 items and were measured in 1 and 0 manner. The full score was 22

† The attitude dimension included 5 items and were measured as ranks (1 to 5). The full score was 30.

‡ The behavior dimension included 9 items and were measured as ranks (1 to 5). The full score was 40.

§ Significantly different from the baseline (*P*<0.05).

### Estimates of intervention effect

Two-level (school, student) models were selected to fit the data after comparing the goodness of fit with three-level (school, grade, student) and four-level (province, school, grade, student) models. Demographic variables (gender, education level of mother and father, left-behind, number of siblings) were insignificant in all models and were excluded. In addition, random effects of intervention, province, grade, knowledge and attitude scores were insignificant and were not introduced to the models.

The results of multilevel DID models are showed in Table 4. Group effects were insignificant in all dimensions, indicating that capability of students in the two groups was balanced at baseline. Time effects were significant, demonstrating that students in control group also performed better compared with baseline. The DID estimators (i.e. group × time) were significant in knowledge and practice dimension but insignificant in attitude. The health education improved students’ knowledge and practice scores by 2.92 (95% CI: 2.06–3.78) and 2.92 (95% CI: 1.37–4.47) respectively. In addition, students from senior grades obtained higher scores; knowledge predicted attitude score; and attitude predicted behavior score. Students from Shaanxi province performed better in all dimensions (especially behavior) than those from Yunnan province.

**Table 4:** Effects estimation by multi-level difference-in-differences models.

	Knowledge			Attitude			Behavior		
	Estimates	SE	*P*	Estimates	SE	*P*	Estimates	SE	*P*
Fixed effect									
Constant (*β*_0_)	9.77	0.31	<0.001	22.06	0.44	<0.001	10.75	1.66	<0.001
Group (*β*_1_)	-0.36	0.34	0.291	0.04	0.32	0.890	-0.81	0.61	0.182
Time (*β*_2_)	1.55	0.30	<0.001	-0.33	0.29	0.261	2.26	0.55	<0.001
Group × Time (*β*_3_)	2.92	0.44	<0.001	0.73	0.42	0.081	2.92	0.79	<0.001
Province (*β*_4_) *	1.72	0.25	<0.001	1.20	0.24	<0.001	7.71	0.45	<0.001
Grade ^†^									
5^th^ (*β*_5_)	1.27	0.22	<0.001	0.76	0.22	<0.001	1.99	0.41	<0.001
6^th^ (*β*_6_)	1.74	0.22	<0.001	0.84	0.23	<0.001	1.33	0.42	0.002
Knowledge score (*β*_7_)	NA			0.27	0.03	<0.001	NA		
Attitude score (*β*_8_)	NA			NA			0.43	0.06	<0.001
Random effect									
School level (*u*_0j_)	1.15	0.40	0.004	0.52	0.25	0.041	1.91	0.47	0.040
Student level (*e*_0ij_)	5.98	0.35	<0.001	6.06	0.33	<0.001	22.20	1.06	<0.001
ICC (%)	16.08			7.85			7.92		
likelihood	3988.31			3955.30			5048.79		

SE: standard error; NA: not applicable; ICC: intra-cluster correlation coefficient

* Yunnan province as reference.

† The 4^th^ grade as reference.

## Discussion

The questionnaire meets psychometric standards. The overall Cronbach’s alpha was 0.84. Among the 54 items tested, 18 presented insufficient discrimination power and were removed. The remaining 36 items yielded a reliable estimate of KAP with respect to nutrition and food safety among primary school students in this specific setting. Through this quasi-experimental program, we found that the nutrition and food safety education significantly improved knowledge and behavior, and not attitude, among primary school students in Grade 4 to 6 in primary schools in poverty-stricken counties of Shaanxi and Yunnan province.

**Fig 4:**
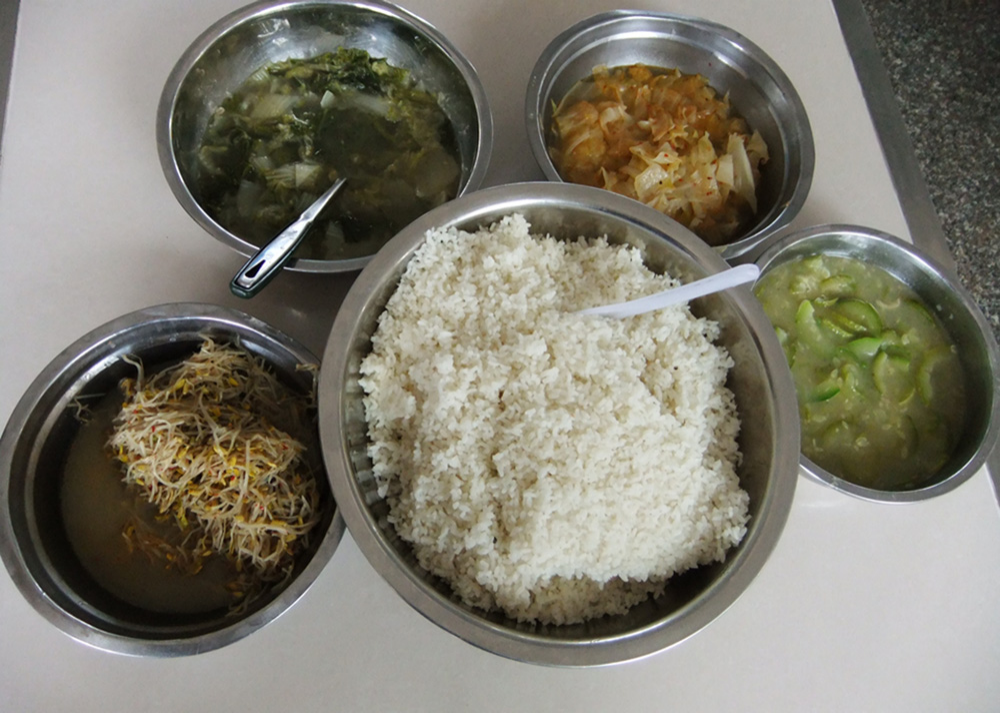
Students’ lunch provided by school. Meal provided by school consisted of four vegetable dishes and rice, and was shared by ten students.

In the development of questionnaire, we applied IRT model. IRT is a family of associated mathematical models that relate latent traits to the probability of responses to items on the assessment [18]. Compared with classical test theory, IRT specifies a nonlinear relationship between binary, ordinal or categorical responses and the latent trait [21], or the capability to answer the questions on nutrition and food safety correctly in current case. A special consideration for the current IRT model was the items with respect to egg, milk and bean products intake in behavior dimension. In remote areas, schools are far from students’ homes and most students live and eat in schools during weekdays. The meals provided by schools were simple, usually 4 vegetable dishes and rice (or noodle) shared by 10 students (Fig 4). Some students brought pickled foods from home and most of them hardly had fresh meats. Shaanxi government established the EGG and MILK PROJECT (mainly funded by local and province governments) in 2009 in order to cope with the undernutrition problem in remote areas: providing each student an egg and 250 ml milk (or soymilk) during schooldays to increase their protein, calcium and vitamin A intake. We removed the related items. The questionnaire with 36 retained items showed good psychometric properties, with high internal consistence and discrimination power.

Demographic characteristics were balanced between intervention and control groups at two time points. Knowledge, attitude, and behavior scores of the intervention group were significantly higher than those of the control, although scores increased in both groups compared with the baseline. The longitudinal difference of scores in control group might be attributed to routine health education, age and cohort effect. The item-specific details of baseline and final investigation were published in previous articles [23, 24].

DID model introduced by Ashenfelter and Card in 1985 [25] has been widely used in econometrics [26, 27]. The method has also been applied in public health researches [28–32] because of its feasibility in treating unbalanced natural trial with or without follow-ups [33]. Owing to the nature of quasi experiment and re-sampling process, DID model was an appropriate method for our situation. However, the data was structured because students were nested in school, and general linear model might not be incorrect because the Gauss-Markov assumption for least square estimation has been violated [34]. Therefore, we combined multilevel model and DID model to perform unbiased estimations, as explained in the following.

Cluster trials are methods that assign social clusters (e.g. schools, communities, factories) instead of individuals into intervention and control groups, and are widely used in effectiveness evaluation of non-treatment intervention such as health education and health policy [35]. Cluster trial will enhance compliance and control contamination of intervention effect between individuals in the same cluster [36]. Besides, it is not feasible to implement such intervention on individual level. Since the students were nested in grades and schools, traditional statistical methods (such as chi-square test for binary indicators and Student’s *t* test for continuous indicators) are not effective in identifying the intra-cluster correlations, and the prerequisite of such hypothesis tests is violated [37].

The observed difference of average scores of knowledge between the two groups was 2.3 in Table 1, while the DID estimator (group × time) was 2.9 according to the multilevel DID model. The DID estimator of behavior was 2.9 but was insignificant for attitude. The average attitude score at baseline was close to 30 (full marks), indicating that many students were positive learners of nutrition knowledge, and they were willing to change unhealthy behaviors before the intervention. Therefore, there might not be much space for making progresses. A statistically significant effect of intervention on behavior was found, although the size of which was small. Vio et al. reported a significant decrease in unhealthy food consumption practice after nutrition education for children aged 7–9 [38]. Similar findings were reported in related papers [12, 13, 39–41], although some of the studies might have methodological differences with respect to statistical techniques. Other nutrition-related intervention studies mainly focused fruit and vegetable consumptions and obesity [42–46], which were important issues in developed countries.

In China, many schools and teachers replace routine health education courses with “major” courses under the pressure of exam-oriented education system [47]. In Chaoyang district of Beijing, 62% of the schools offered health education for students in 2005 [48]. In rural areas of Gansu province, only 15% of the schools opened health education courses [49]. In addition, traditional health education primarily focuses on child and adolescence health and safety education. Nutrition and food safety knowledge are very limited in these courses. Our intervention initiated an opportunity for young children in remote areas of China to learn nutrition and food safety knowledge that was highly related to their daily life, and helped them to change their attitude and behaviors. There are several implications of our study. First, although the one-year intervention in our study is effective, it is insufficient in facilitating substantial knowledge improvement and behavior change among students. Long-term and persistent intervention should be implemented in the future. Second, the study serves as an example of appropriate statistical techniques for assessments of questionnaire and intervention effectiveness. IRT, the modern test theory, is effective in the assessment of questionnaire / scale reliability, and should be widely applied in health education settings. DID model is efficient and non-biased in evaluating the effectiveness of quasi-experiments without repeatedly measured data.

There were limitations in our study. First, we did not conduct follow-ups at individual level and perform repeated measures, because we spent one year on targeted textbooks designing according to the baseline information. Re-sampling might decrease the power of statistical inference and have impact on the external validity of our research conclusions, although our samples were randomly selected, and all demographic characteristics were balanced between two groups. Second, our sample size was not large. Distances between two schools varied from 20 kilometers to more than 100 kilometers (most of the distances exceeded 50 kilometers) and all of them were located in hazardous mountainous areas. It took half a day or longer driving from one school to another. Meanwhile, the number of students in different schools varied from dozens to hundreds, and we had to compromise the intra-school sample size to around 35 during data collections. Finally, we could not identify students who had not received educational courses due to absenteeism, sickness or other uncontrolled conditions during the intervention. We assumed that all students in intervention group completed the course.

There are several policy suggestions with respect to our findings. First, nutrition and food safety education should be emphasized in schools of China instead of the exam-oriented teaching pattern. Second, nutrition and food safety education should be a required curriculum in training primary and middle school teachers. Third, textbooks and teaching materials for nutrition and food safety are non-existent in China. They should be designed for students at different age and in different areas, respectively, in order to achieve specific goals. Last, nutrition and food safety education should be integrated into the system of health education for students. Our study can provide experiences for the targeted health education in remote areas of China.

In conclusion, we conducted a quasi-experimental intervention on improving the knowledge, attitude and practice of nutrition and food safety for primary students in poverty-stricken counties of China, aiming to develop and assess the questionnaire and evaluate the effectiveness of the intervention in ameliorating the status of malnutrition. We designed a well targeted textbook for students in grade 4–6 and successfully improved their nutrition and food safety knowledge and behavior, compared with the control group. Long-term, large-scale and randomized trials are needed to test the effectiveness and benefits of nutrition-related health education for students in remote and poorly developed regions.

## Supporting Information

S1 Dataset Anonymized data of baseline and end point survey. (XLS)
